# Diagnostic accuracy of anti-integrin αvβ6 in ulcerative colitis: a diagnostic meta-analysis

**DOI:** 10.1007/s00535-025-02319-8

**Published:** 2025-11-13

**Authors:** Mudasar Nisar, Abdul Rafeh Awan, Abdullah Ahmad, Hira Saleem, Meher Ayyazuddin, Rabia Javed, Ali Raza Khan, Muhammad Ahmad Nadeem, Maryam Abbas Malik, Amir Humza Sohail, Abu Baker Sheikh

**Affiliations:** 1 Services Institute of Medical Sciences, Lahore, Pakistan; 2 Nishtar Medical University, Multan, Pakistan; 3 CMH Lahore Medical College, Lahore, Pakistan; 4 Jinnah Hospital, Lahore, Pakistan; 5 Department of Internal Medicine, Carepoint Bayonne Medical Center, Bayonne, NJ USA; 6 Department of Internal Medicine, Trinity Health Livonia Hospital/Wayne State University Program, Detroit, MI USA; 7 Digestive Diseases Institute, Clevelenad Clinic, Cleveland, OH USA; 8 Department of Surgical Oncology, University of New Mexico, Albuquerque, NM USA; 9 Department of Internal Medicine, University of New Mexico, Albuquerque, NM USA

**Keywords:** Ulcerative colitis, Anti-integrin αvβ6, Sensitivity, Specificity, Diagnostic meta-analysis

## Abstract

**Background:**

Ulcerative colitis (UC) presents with diagnostic challenges due to symptom overlap with other gastrointestinal (GI) disorders. Recent studies identify anti-integrin αvβ6 autoantibodies as a promising biomarker for UC. This meta-analysis aims to evaluate the diagnostic accuracy of anti-integrin αvβ6 antibodies in distinguishing UC from healthy individuals and other GI diseases.

**Methods:**

We conducted a systematic literature search of PubMed, Scopus, and Embase up to February, 2025, following PRISMA guidelines. Studies assessing the diagnostic performance of anti-integrin αvβ6 antibodies in UC patients were included. A bivariate random-effects model was used to pool sensitivity and specificity estimates using STATA. Forest plots and SROC curves were generated. Meta-regression and interaction analyses explored the influence of covariates such as control group type, age group, and geographic region on diagnostic performance. Risk of bias was assessed using tool QUADAS-2. Post hoc analyses were conducted to assess the impact of cut-off thresholds and ELISA platforms on the diagnostic performance of anti-integrin αvβ6 antibodies.

**Results:**

Six studies comprising 3887 participants (1904 UC patients) were included in meta-analysis. The pooled sensitivity and specificity of anti-integrin αvβ6 for UC across all comparator groups were 83% (95% CI: 0.70–0.91) and 93% (95% CI: 0.88–0.97), respectively. Diagnostic performance remained consistent across control types for sensitivity but varied significantly for specificity, especially when Crohn’s disease was used as a comparator (81%; 95% CI: 0.75–0.86). Multivariate meta-regression identified patient age, geographic region, and control group type as significant modifiers of specificity. Interaction models further confirmed a combined influence of these factors on diagnostic performance. Post hoc analyses revealed that sensitivity remained stable across thresholds (2SD: 0.65, 3SD: 0.87), while specificity varied significantly depending on cut-off values (2SD: 0.89, 3SD: 0.92) and ELISA methodology (0.92 vs. 0.83).

**Conclusions:**

Our meta-analysis demonstrates that anti-integrin αvβ6 antibodies exhibit high diagnostic accuracy for UC, with consistent sensitivity and specificity. Their performance is influenced by patient demographics and study region, suggesting the need for tailored diagnostic criteria in clinical settings.

**Supplementary Information:**

The online version contains supplementary material available at 10.1007/s00535-025-02319-8.

## Introduction

Ulcerative colitis (UC) and Crohn’s disease (CD) are the two primary forms of inflammatory bowel disease (IBD), a chronic immune-mediated disorder of the gastrointestinal tract. UC typically begins in the rectum and extends proximally in a continuous manner, characterized by a relapsing-remitting course [1]. Disruption of the intestinal epithelial barrier plays a central role in UC pathogenesis either as a consequence of inflammatory mediators or as a primary defect that triggers immune activation and perpetuates mucosal injury [2]. The global burden of UC is substantial, with high incidence in Western nations and rising prevalence in newly industrialized regions, suggesting both genetic predisposition and environmental influences in disease pathogenesis [2].

The clinical presentation of UC typically involving diarrhea, rectal bleeding, abdominal pain, and systemic symptoms overlaps substantially with other gastrointestinal disorders, including Crohn’s disease, infectious colitis, and NSAID enteropathy, complicating early diagnosis. While consensus guidelines exist, there is no single diagnostic test for UC. Diagnosis typically relies on an integrated assessment of clinical presentation, serologic markers, stool biomarkers, endoscopic findings, and histopathology [3]. Biomarkers such as CRP, fecal calprotectin (fCP), and serum inflammatory markers (e.g., anemia, hypoalbuminemia) are commonly employed to support diagnosis, differentiate IBD from functional GI disorders, and monitor disease activity [4].

Emerging evidence has identified anti-integrin αvβ6 autoantibodies as a promising serologic biomarker for the diagnosis of UC [5, 6]. Integrin αvβ6 is a heterodimer expressed on epithelial cells where it functions to activate transforming growth factor-β1 (TGF-β1) and modulate innate immune responses in lungs, skin, and GI tract [7] to play a crucial role in maintaining epithelial barrier functions [8]. Autoantibodies can interfere with the normal function of integrin αvβ6, potentially leading to compromised epithelial barrier function and increased susceptibility to inflammation. Notably, longitudinal studies suggest that αvβ6 autoantibodies may precede clinical UC diagnosis by up to a decade, indicating potential utility in preclinical detection and risk stratification [5, 6]. Studies across different regions have identified anti-integrin αvβ6 to have high sensitivity and specificity in diagnosis UC against other GI diseases [5, 9].

This meta-analysis aims to systematically assess the diagnostic accuracy of anti-integrin αvβ6 antibodies for ulcerative colitis, with subgroup analyses exploring the influence of control type, age, and geographic region on test performance.

## Methodology

### Search strategy

This meta-analysis was conducted in accordance with the Preferred Reporting Items for Systematic Reviews and Meta-Analyses (PRISMA) guidelines [10] and the methodological framework outlined by the Cochrane Collaboration [11]. A comprehensive literature search was performed on February 19, 2025, across three major electronic databases: PubMed, Scopus, and Embase.

The following search string was employed:

*(“anti-integrin αvβ6” OR “anti-integrin avb6” OR “αvβ6 antibody” OR “avb6 antibody” OR “integrin αvβ6” OR “integrin avb6” OR “αvβ6 inhibitor” OR “avb6 inhibitor” OR “integrin inhibitor” OR “integrin αvβ6 blockade”) AND (“ulcerative colitis” OR “UC” OR “inflammatory bowel disease” OR “IBD” OR “colitis ulcerosa” OR “chronic ulcerative colitis” OR “inflammatory colitis” OR “intestinal inflammation”)*

**Fig. 1:**
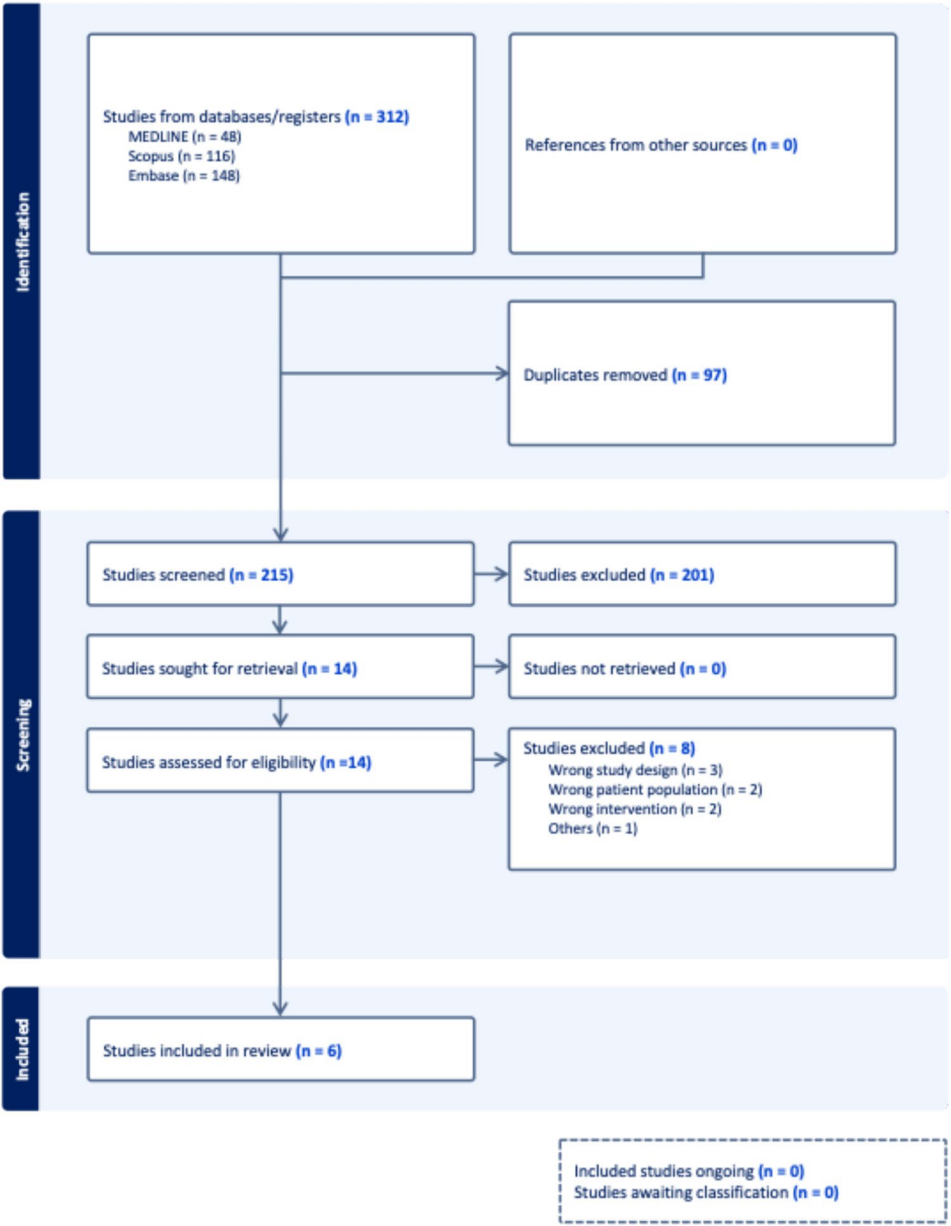
PRISMA flow diagram detailing the systematic review process for studies assessing anti-integrin αvβ6 antibodies in ulcerative colitis, from initial search through inclusion

No restrictions were applied regarding language, publication date, or geographic location. The study selection process and results of the search are detailed in the PRISMA flow diagram (Fig. 1).

### Study selection and eligibility criteria

Studies were considered eligible for inclusion if they met the following criteria:

- Conducted on human subjects presenting with clinical features of inflammatory bowel disease (IBD)
- Reported diagnostic performance metrics for αvβ6 antibodies, including sensitivity, specificity, or area under the receiver operating characteristic curve (AUC)
- Included participants of any age group
- Employed a study design such as randomized controlled trials (RCTs), clinical trials, or observational studies (prospective or retrospective cohorts)

Studies were excluded if they met any of the following conditions:

- Animal-based research
- Exclusively included patients with Crohn’s disease (CD) without reference to ulcerative colitis (UC)
- Included patients with a diagnosis or symptoms of primary sclerosing cholangitis (PSC)
- Represented non-original literature (e.g., meta-analyses, narrative/systematic reviews, letters to the editor, or conference abstracts)

Following the database search, all retrieved records were manually reviewed to eliminate duplicates. Two independent reviewers (MN and ARA) then conducted a two-phase screening process. First, studies were screened by title and abstract to identify potentially relevant articles. This was followed by a full-text review of shortlisted articles to determine final eligibility based on predefined inclusion criteria. Any disagreements between reviewers were resolved by consultation with a third independent author (AHS). In addition, the reference lists of all included studies were manually reviewed to identify any further eligible articles that may have been missed during the initial search.

### Data extraction and quality assessment

After final inclusion, data extraction was performed independently by two reviewers (RJ and AA) using a structured Excel-based data collection form. Extracted variables included baseline study characteristics, patient demographics, and diagnostic performance metrics. Where available, raw data including true positives (TP), true negatives (TN), false positives (FP), and false negatives (FN) were directly extracted. In cases where such values were not explicitly reported, they were derived from the sensitivity and specificity metrics provided in the original studies.

The methodological quality and risk of bias in the included studies were assessed using the QUADAS-2 tool [12]. This tool evaluates the integrity of diagnostic research across four domains: patient selection, index test, reference standard, and flow and timing. Each domain is assessed for potential bias and concerns regarding applicability. Judgments are categorized as “low,” “high,” or “unclear” risk. The tool employs visual representations, typically using color-coded bar graphs and summary plots, to illustrate the distribution of risk of bias and concerns regarding applicability across all included studies.

### Statistical analysis

All statistical analyses were conducted using STATA version 17.0 [13], employing the *metadta* module to perform a bivariate random-effects meta-analysis of diagnostic test accuracy. True positive (TP), false positive (FP), false negative (FN), and true negative (TN) values were either directly extracted or reconstructed from reported sensitivity and specificity metrics. Considering the variability in ELISA thresholds and kits across studies, the equivalence of raw positivity rate was not assumed. A bivariate random-effects model was applied, which analyzed sensitivity and specificity together, allowing valid pooling (even in the event of studies using different index test thresholds). Summary estimates of sensitivity and specificity, along with their corresponding 95% confidence intervals (CIs), were calculated, with statistical significance set at a p value of less than 0.05. Heterogeneity was assessed using the *I*^2^ statistic [14]. Forest plots of pooled sensitivity and specificity as well as summary receiver operating characteristic (SROC) curves were generated for visual representation of test performance.

To explore sources of heterogeneity and assess the impact of key moderators, a series of meta-regression models were constructed. Covariates included control group type (healthy, diseased, Crohn’s disease), age group (adults vs. pediatric), and geographic region (Europe, Japan, United States). Independent meta-regression models were first fitted to evaluate the effect of control-type alone, as well as in combination with age group (adult vs pediatric) and study region (Europe vs Japan vs US), on test sensitivity and specificity. Control type (healthy, diseased, Crohn’s disease) served as the primary covariate, with age and region included as additional moderators in separate models. Subsequently, to assess potential effect modification, we fitted extended models incorporating interaction terms, specifically control × age, control × region, and control × age × region. Wald-type tests were used to assess the significance of individual predictors, while likelihood ratio (LR) tests were employed to compare nested models (with and without interaction terms) to determine if the interaction terms significantly improved model fit. All models were estimated using a bivariate random-effects framework. Between-study heterogeneity was assessed using tau-squared (τ^2^) and I^2^ statistics (Table 1).

**Table 1:** Study and patient characteristics

Study ID	Cohorts	Study design	Sample size				Male			Age at time of sample (mean)	Age at diagnosis (mean)
			Total	UC	OGD	HC	UC	OGD	HC		
Okabe 2025	–	Multicenter cohort study	2243	1241	1002; CD: 796, OGD: 206	NA	673	645	NA	UC: 46.5 ± 16.4 CD: 41.6 ± 14.1 OGD: 55.5 ± 17.6	UC: 35.0 ± 15.5 CD: 28.2 ± 13.0 OGD: 49.3 ± 20.3
Marafini 2023	–	Prospective observational study	273	108	CD: 103	62	54	61	NR	UC: 46.2 ± 11.9 CD: 47.5 ± 11.9	NR
Livanos 2023	PREDICTS	Cohort study	164	82	NA	82	79	NA	79	UC: 32.0 ± 6.4 HC: 32.6 ± 5.6	UC: 32.0 ± 6.4 HC: 32.6 ± 5.6
	CCC-GEM		61	12		49	6		24	UC: 17.3 ± 7.5 HC: 17.7 ± 7.1	NR
	COMPASS		109	55		54	28		31	NR	UC: 31.3 ± 12.4 HC: 46.3 ± 15.9
	OSCCAR		158	104		54	42		31	NR	UC: 36.8 ± 20.1 HC: 46.3 ± 15.9
Muramoto 2022	–	Cross-sectional diagnostic accuracy study	329	131	130; CD: 95 OGD: 10 PID: 25	68	65	80	27	NR	NR
Rydell 2022	–	Cross-sectional study	273	59	138; CD: 38 IBS: 100	76	33	47	NR	UC: 43.3 ± 13.2 CD: 44 ± 13.6 IBS: 36.8 ± 7.8	NR
Kuwada 2021	–	Cross-sectional diagnostic accuracy study	277	112	NA	165	59	NR	97	NR	NR

*UC* Ulcerative colitis, *CD* Crohn’s disease, *HC* Healthy control, *OGD* Other GI diseases, *NA* Not applicable, *NR* Not reported

To further assess methodological sources of heterogeneity, we performed post hoc meta-regression analyses incorporating (i) cut-off rule (2SD/95th percentile vs. 3SD from control means; Table 2) and (ii) ELISA platform (the Kuwada-referenced method, used in most studies, vs. alternative platforms; Table 2). Models were estimated both independently and in combination with control group type or study region. Because region and cut-off were perfectly collinear (all European studies exclusively applied 2SD/95th-percentile thresholds), these joint models did not converge. Accordingly, region and cut-off effects were examined separately.

**Table 2:** Summary of ELISA kits and diagnostic cut-off values used for anti-integrin αvβ6 antibody detection in ulcerative colitis studies

Study ID	Region	ELISA kit used	Definition of cut-off value	Cut-off value for diagnosis (U/ml)
Okabe 2025	Japan	Medical and Biological Laboratories Co. Ltd., Tokyo, Japan	The mean+3 standard deviations (SDs) of 83 serum samples from healthy volunteers as determined by the manufacturer	1.64
Marafini 2023	Europe	Bethyl Laboratories, Montgomery, TX, USA	The cut-off for positivity was defined as the 95th percentile of the AU values of HC.	NR
Livanos 2023	United States	Bethyl Laboratories, Montgomery, TX, USA	The cut-off for positivity was defined as greater than the mean HC plus 3 SD	0.99
Muramoto 2022	Japan	Bethyl Laboratories, Montgomery, TX, USA	The mean plus 3 standard deviations of the sera of non-enterocolitis patients	0.25
Rydell 2022	Europe	Bethyl Laboratories, Montgomery, TX, USA	Cut-off for positive results was defined as the 95th percentile of the AU values of 76 Red Cross blood donors (i.e., the HC subjects).	1
Kuwada 2021	Japan	Bethyl Laboratories, Montgomery, TX, USA	The cut-off was defined as the mean plus 3 SDs of the control sera	0.25

## Results

### Literature search synthesis

Our systematic search identified 312 records from the database(s). Following the removal of 97 duplicates, 215 unique records underwent title and abstract screening. Of these, 14 studies were deemed potentially eligible and proceeded to full-text retrieval and assessment. Ultimately, 6 studies [5, 9, 15–18] satisfied all inclusion criteria and were incorporated into the meta-analysis. The study selection process is detailed in the PRISMA flow diagram (Fig. 1).

### Study characteristics and quality assessment

Three of the included studies were cross-sectional diagnostic accuracy studies, two were cohort studies and one was a prospective observational study. One study (Livanos et al. [15]) analyzed four independent cohorts, of which two met the inclusion criteria and were included in this analysis.

**Fig. 2:**
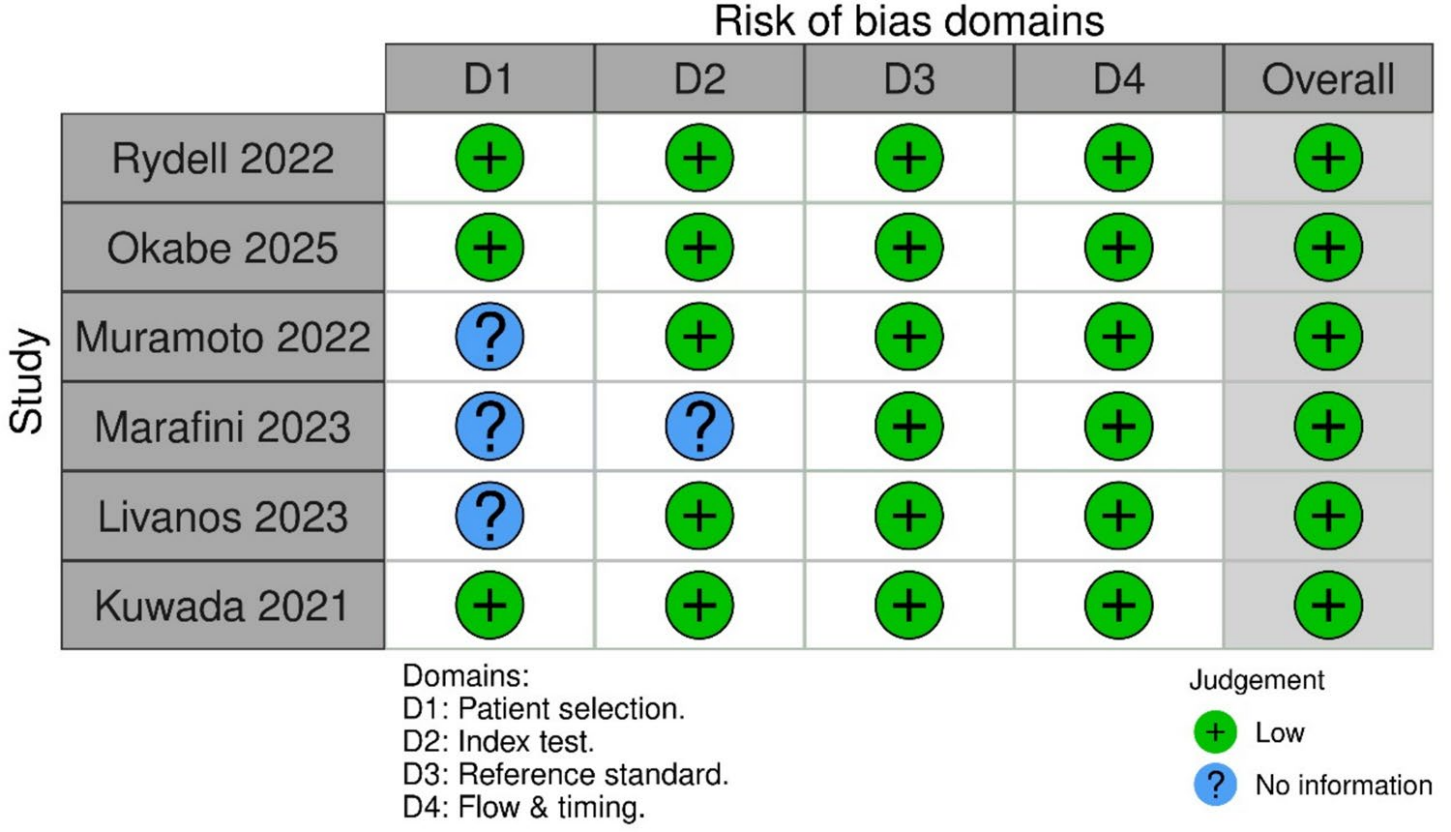
QUADAS-2 risk of bias summary plot showing individual judgments across the four for each included study

**Fig. 3:**
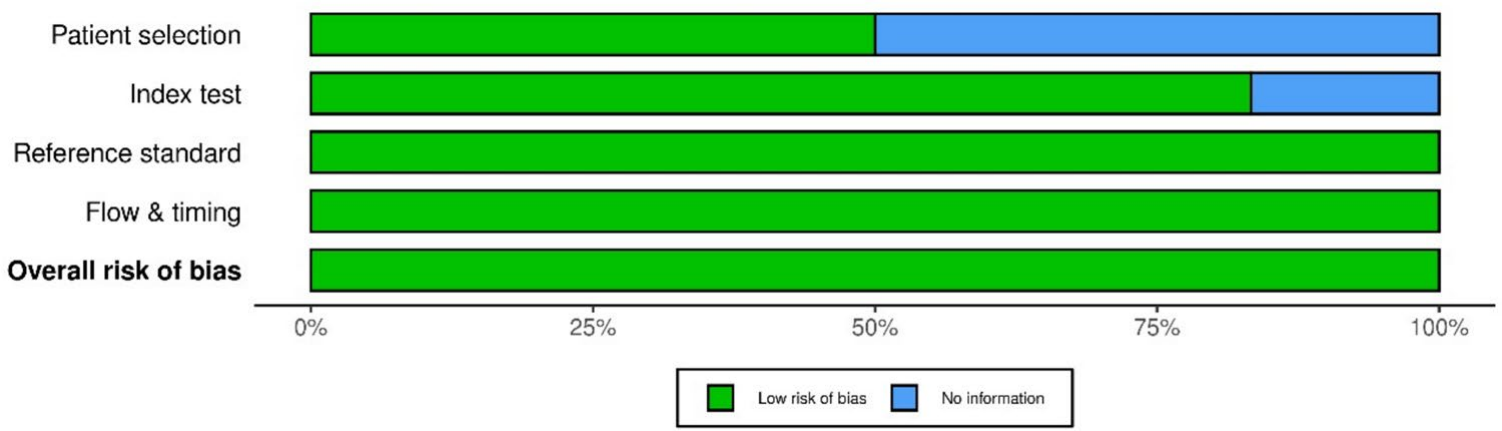
QUADAS-2 domain-level risk of bias bar chart summarizing the proportion of studies with low, high, or unclear risk of bias across each domain

All six studies were found to be of high quality with low overall risk of bias, based on quality assessment via QUADAS-2. Aside from the patient selection domain, where three studies had unclear risk due to limited reporting, all studies demonstrated low risk in the remaining domains (index test, reference standard, and flow and timing). Figures 2 and 3 summarize the individual and domain-level risk of bias across included studies.

### Baseline characteristics

This meta-analysis incorporated data from 3887 participants across the included studies. Among these, 1904 patients were diagnosed with ulcerative colitis (UC). UC cohorts demonstrated a consistent male predominance, with males representing 54% to 97% of UC patients in studies reporting gender. Control groups comprised healthy individuals (HC; reported in 5 studies) and patients with other gastrointestinal disorders (OGD; reported in 4 studies), predominantly Crohn’s disease. Age at the time of sample collection was reported in 5 studies, while age at UC diagnosis was reported in 4 studies. Where both were reported, the age at UC diagnosis was consistently lower than the age at sampling. Smoking status was infrequently reported across the studies (Table 1).

### Diagnostic accuracy analysis

**Fig. 4:**
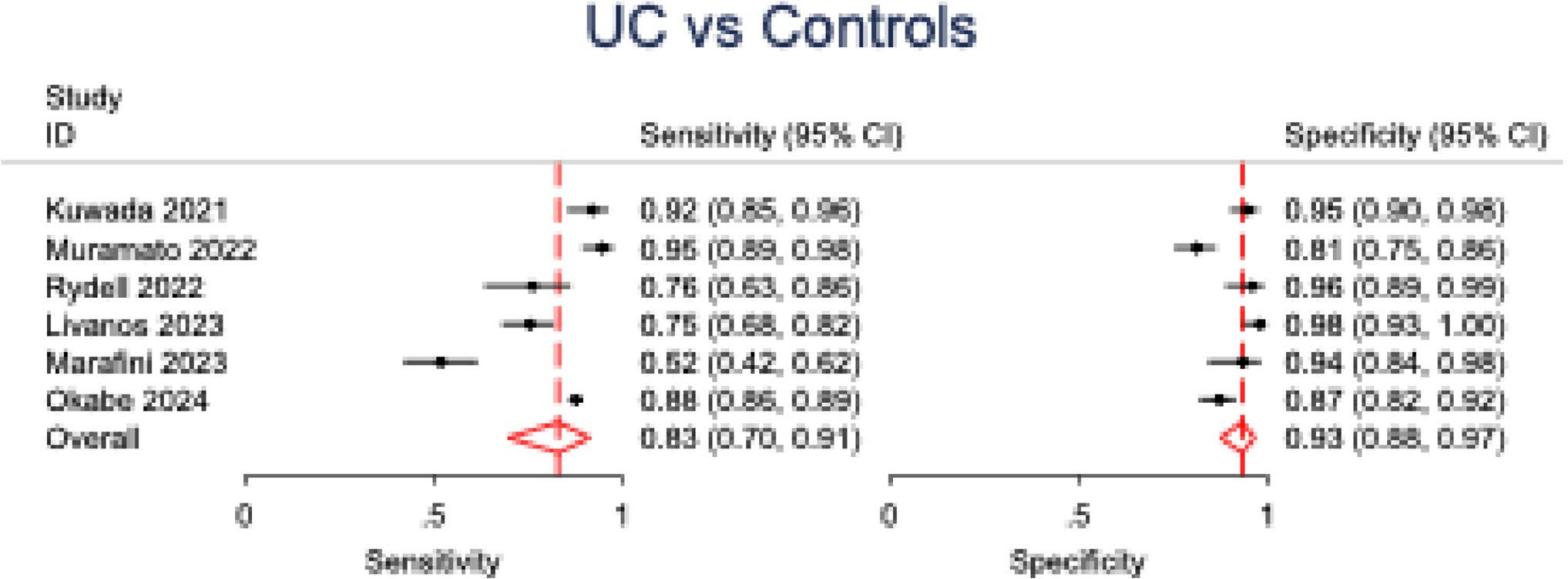
Forest plot of pooled sensitivity and specificity for anti-integrin αvβ6 antibodies in diagnosing ulcerative colitis versus all control groups

**Fig. 5:**
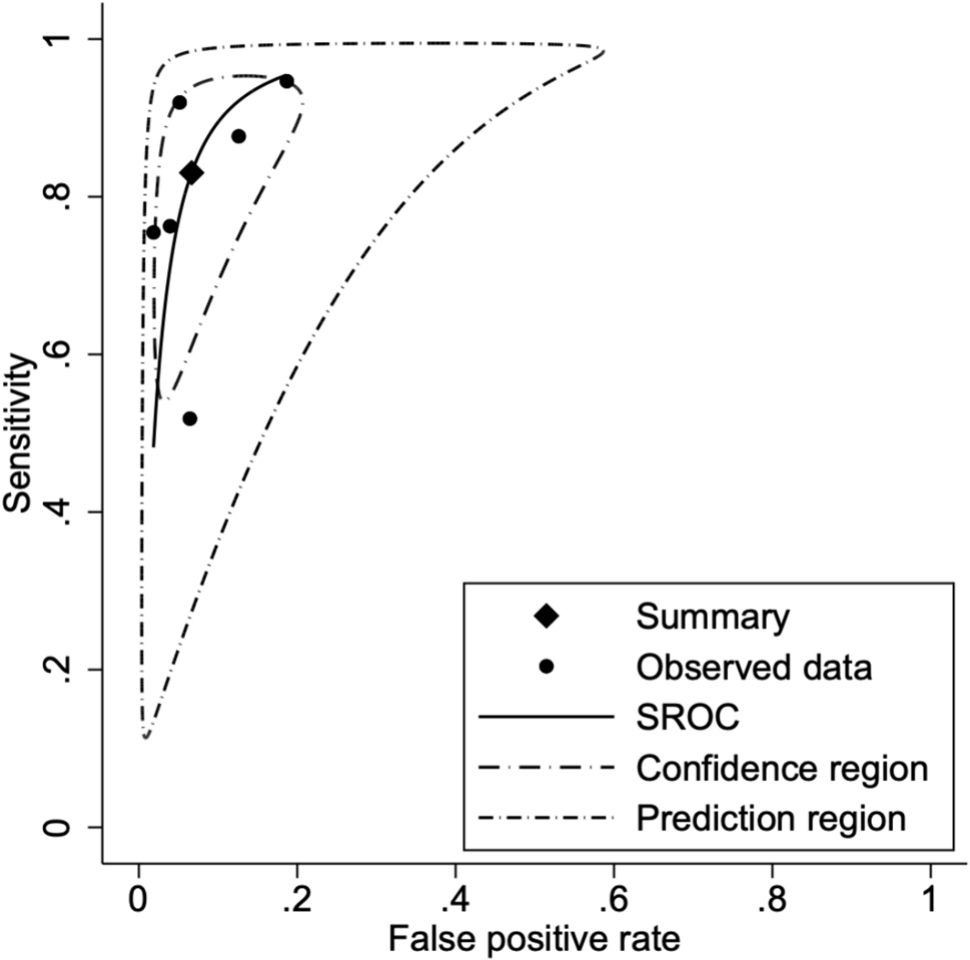
SROC curve for anti-integrin αvβ6 antibodies in distinguishing ulcerative colitis from all control groups

In the pooled analysis of six studies evaluating anti-integrin αvβ6 antibody accuracy for ulcerative colitis detection across all comparator groups, a bivariate random-effects meta-analysis model demonstrated an overall sensitivity of 83% (95% CI: 0.70–0.91; *p* < 0.001; *I*^2^ = 91%) and overall specificity of 93% (95% CI: 0.88–0.97; *p* < 0.001; *I*^2^ = 73%). The summary receiver operating characteristic (SROC) curve indicated robust discriminatory ability, with coherent confidence and prediction regions around the summary point. Forest plots and SROC curves for UC versus all controls are presented in Figs. 4 and 5, respectively.

**Fig. 6:**
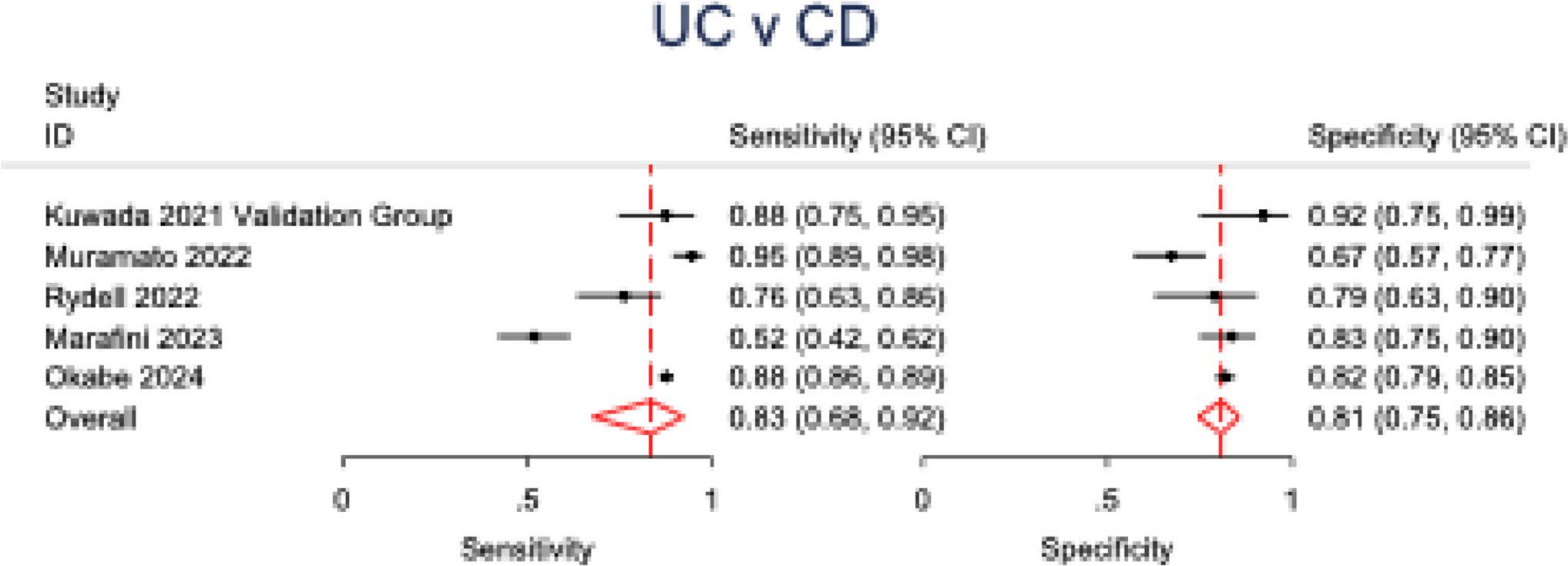
Forest plot of pooled sensitivity and specificity for anti-integrin αvβ6 antibodies in differentiating ulcerative colitis from Crohn’s disease

**Fig. 7:**
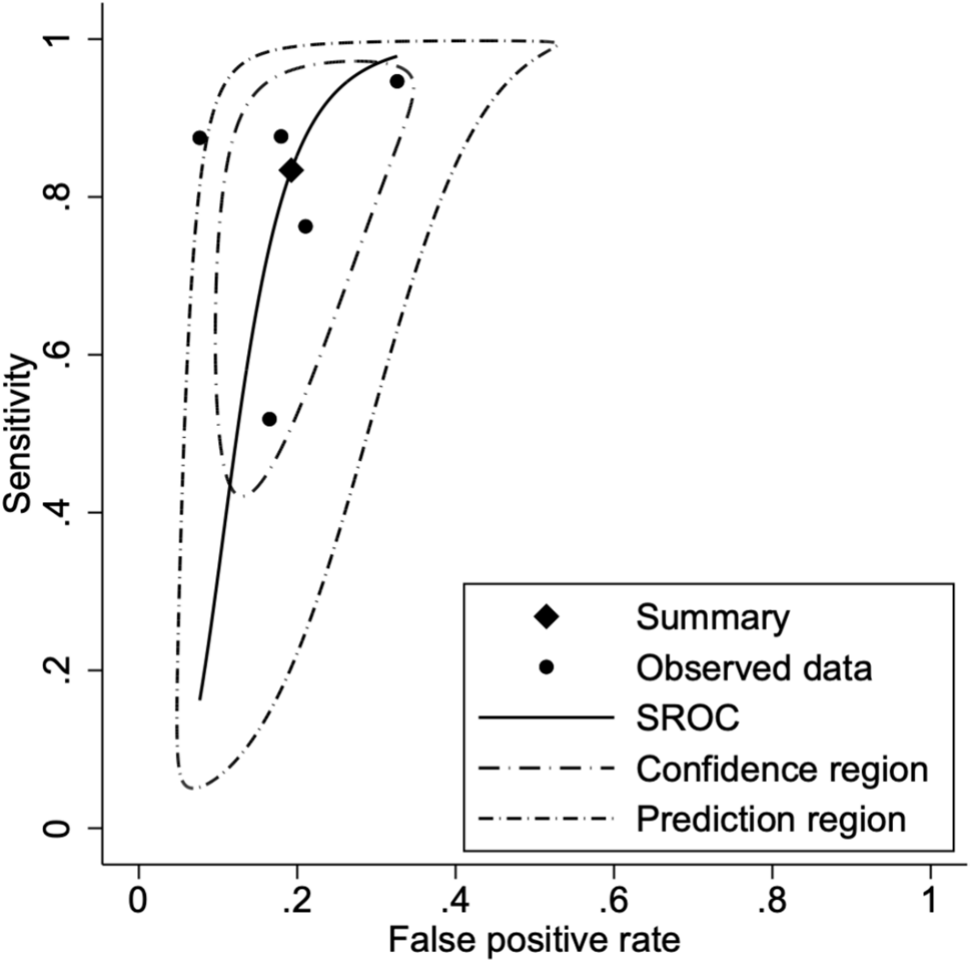
SROC curve evaluating the diagnostic performance of anti-integrin αvβ6 antibodies in distinguishing ulcerative colitis from Crohn’s disease

**Fig. 8:**
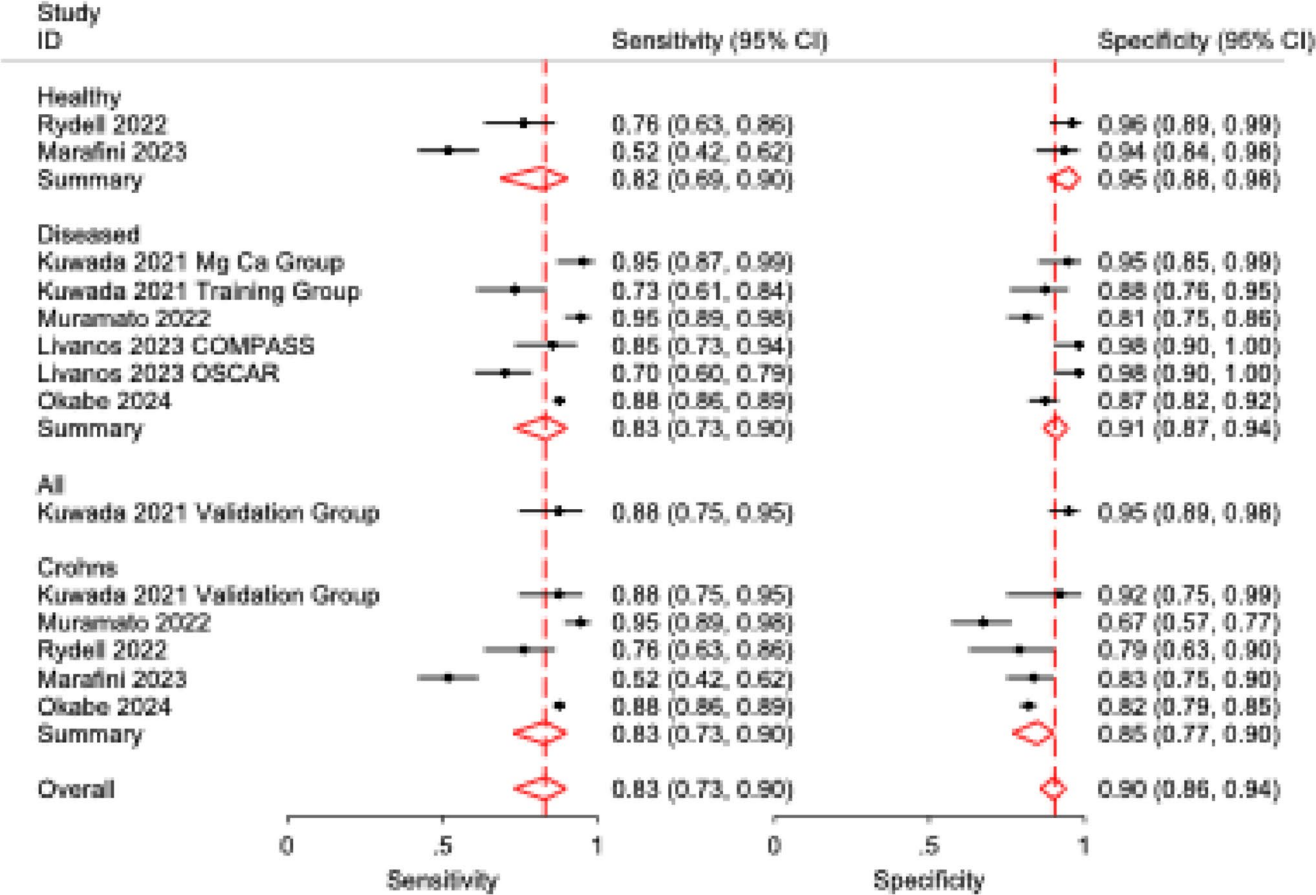
Forest plot of sensitivity and specificity across all comparator subgroups (healthy controls, diseased controls, and Crohn’s disease) for anti-integrin αvβ6 antibody performance

Five studies were analyzed to evaluate the discriminatory capacity of anti-integrin αvβ6 antibodies for ulcerative colitis versus Crohn’s disease. UC was found to have a sensitivity of 83% (95% CI: 0.68–0.92; *p* < 0.001; *I*^2^ = 88%) and specificity of 81% (95% CI: 0.75–0.86; *p* < 0.001; *I*^2^ = 44%) when compared to CD. Similar to UC versus all controls, the SROC curve for UC versus CD showed good discriminatory powers. Further subgroup stratification revealed: when compared to healthy controls (2 studies), sensitivity was 82% (95% CI: 0.69–0.90) with specificity of 95% (95% CI: 0.88–0.98); while against all diseased cohorts (5 studies), sensitivity was 83% (95% CI: 0.73–0.90) with specificity of 91% (95% CI: 0.87–0.94) (Table 3). Forest plots and SROC curves for UC versus CD are presented in Figs. 6 and 7, respectively, while the forest plot for all subgroups is presented in Fig. 8.

**Table 3:** Overall pooled sensitivity and specificity of αvβ6 antibody for differentiating ulcerative colitis (UC) from controls and from Crohn’s disease

Test	Sensitivity			Specificity		
	Sensitivity (95% CI)	*p* value	*I*^2^	Specificity (95% CI)	*p* value	*I*^2^
UC vs. controls	0.83 (0.70–0.91)	< 0.001	91.00%	0.93 (0.88–0.97)	< 0.001	73.83%
UC vs. Crohn’s	0.83 (0.68–0.92)	< 0.001	88.74%	0.81 (0.75–0.86)	< 0.001	44.61%

*p*-values <0.05 were considered statistically significant

Heterogeneity across studies is reported using the *I*2 statistic

#### Independent meta-regression

To identify potential contributors to between-study heterogeneity in the diagnostic performance of the αvβ6 antibody for ulcerative colitis (UC), we first performed a series of independent meta-regression analyses, considering each covariate separately and in combination. Univariate meta-regression based on control group type (Table 4) revealed that specificity differed significantly across control subgroups (Wald χ^2^ = 11.03; df = 3; *p* = 0.0115), with the lowest specificity observed when Crohn’s disease was used as the comparator. However, relative sensitivity remained statistically similar across subgroups (χ^2^ = 0.18; *p* = 0.98), indicating that αvβ6 maintains stable sensitivity regardless of control group type.

**Table 4:** Relative diagnostic performance of αvβ6 antibody in differentiating ulcerative colitis from different control groups (healthy control as reference)

Test	Relative sensitivity			Relative specificity		
	Sensitivity (95% CI)	*p* value	*I*^2^	Specificity (95% CI)	*p* value	*I*^2^
Healthy controls	1.00 (Reference)			1.00 (Reference)		
Diseased controls	1.02 (0.93–1.12)	0.71	–	0.96 (0.91–1.01)	0.10	–
All controls	1.03 (0.85–1.26)	0.74	–	0.99 (0.90–1.08)	0.74	
Crohn’s disease	1.01 (0.93–1.10)	0.74	–	0.89 (0.83–0.95)	< 0.001	

Test	Chi^2^	df	*p* value	Chi^2^	df	*p* value
*Wald-type test for nonlinear hypothesis*						
Control	0.18	3	0.9807	11.03	3	0.0115

*p*-values <0.05 were considered statistically significant

Univariate independent meta-regression assessing relative sensitivity and specificity of αvβ6 using healthy controls as the baseline comparator. Wald-type test used to assess overall difference across subgroups

We then conducted multivariable independent meta-regression models by pairing control type with either age group or region of study (Table 5). In the model adjusting for age, specificity was significantly affected by both control type (χ^2^ = 7.40; *p* = 0.01) and age group (χ^2^ = 27.30; *p* < 0.05), while sensitivity remained unaffected (*p* = 0.53). In the model adjusting for region, both control type (χ^2^ = 10.50; *p* = 0.01) and study region (χ^2^ = 27.17; *p* < 0.001) were again significant determinants of specificity. The full model incorporating both covariates further confirmed a robust association between these factors and variation in specificity (χ^2^ = 30.79; *p* < 0.001). These findings indicate that the diagnostic specificity of αvβ6 antibody is more sensitive to clinical context including population demographics and geographical setting than sensitivity.

**Table 5:** Multivariate independent meta-regression of αvβ6 diagnostic accuracy by type of control, age group, and region of study

Variable	Sensitivity			Specificity		
	Chi^2^	df	*p* value	Chi^2^	df	*p* value
*Meta-regression by type of control and age of participants*						
Control (healthy control as standard)	3.02	1	0.08	7.40	1	0.01
Age (adults as standard)	0.28	3	0.96	27.30	3	< 0.001
Full model	3.15	4	0.53	30.79	4	< 0.001
*Meta-regression by type of control and region of study*						
Control (healthy control as standard)	6.53	2	0.04	10.50	2	0.01
Region (Europe as standard)	0.04	3	1.00	27.17	3	< 0.001
Full model	6.82	5	0.23	34.80	5	< 0.001

*p*-values <0.05 were considered statistically significant

Leave-one-out likelihood ratio test (LR test) assessing the contribution of each covariate (control type, age, region) to between-study heterogeneity in sensitivity and specificity for UC diagnosis. Full model includes all covariates

Between-study heterogeneity, evaluated using Tau^2^ and LR tests (Supplementary Table 1), also declined when multiple covariates were incorporated, supporting the role of age and region as modifiers of diagnostic accuracy. Specifically, the generalized Tau^2^ dropped from 0.15 in the univariate control-type model to 0.00 in both multivariate models, with corresponding reductions in Tau^2^ for specificity and sensitivity. This reduction suggests improved model fit and a partial explanation for observed heterogeneity.

#### Interaction meta-regression

To further investigate whether the effects of covariates interact in modifying diagnostic accuracy, we conducted meta-regression models incorporating interaction terms between control type, age group, and region (Table 6). In univariate interaction analyses, neither the control*age nor control*region terms significantly influenced sensitivity or specificity. The interaction between control*age did not yield statistically significant Wald or LR test values for either outcome (Sensitivity: Wald χ^2^ = 0.00, *p* = 0.9632; LR test χ^2^ = 0.00, *p* = 0.99, Specificity: Wald χ^2^ = 0.00, *p* = 0.9632; LR test χ^2^ = 0.00, *p* = 0.99). The interaction between control*region showed borderline significance for specificity (Sensitivity: LR test χ^2^ = − 0.02, *p* = 1.00; Specificity: LR test χ^2^ = 3.48, *p* = 0.06).

**Table 6:** Interaction meta-regression of αvβ6 antibody accuracy by control type, age group, and region

Variable	Sensitivity					Specificity				
	RR (95% CI)	Wald χ^2^	*p* value	Leave one out LR χ^2^	*p* value	RR (95% CI)	Wald χ^2^	*p* value	Leave one out LR χ^2^	*p* value
Control vs. Crohn’s	–	–	–	0.00	0.97	–	–	–	20.91	< 0.001
Age (adults and controls as standard)	1.00 (0.96–1.04)	0.00	0.9632	Age*control: 0.00	0.99	0.89 (0.85–0.92)	0.87	0.3502	Age*control: 0.38	0.54
Region (Europe and controls as standard)	0.61 (0.56–0.65)	22.47	< 0.001	Region*control: − 0.02	1.00	0.54 (0.52–0.55)	370.70	< 0.001	Region*control: 3.48	0.06
*Multivariable meta-regression*										
Age (adults and controls as standard)	–	3.75	0.0528	Age*control: 0.00	0.98	–	4.93	0.0264	Age*control: 2.00	0.16
Region (Europe and controls as standard)	–	37.24	< 0.001	Region*control: 0.00	0.99	–	476.81	< 0.001	Region*control: 0.04	0.84
Full model (age × region × control)	0.60 (0.57–0.64)	–	–	Age*region*control: 8.96	0.18	0.54 (0.53–0.55)	–	–	Age*region*control: 37.39	< 0.001

*p*-values <0.05 were considered statistically significant

Interaction meta-regression evaluated whether diagnostic performance varied across combinations of control type, age group, and study region. Pairwise and three-way interaction terms were tested using Wald and likelihood ratio tests, with results reported as relative risks and heterogeneity statistics

In the multivariable interaction model incorporating age, control, and region, interaction effects did not significantly influence sensitivity (All LR χ^2^ = 8.96, *p* = 0.18). However, for specificity, the joint inclusion of interaction terms significantly improved model fit (All LR χ^2^ = 37.39, *p* < 0.001), suggesting that specificity is influenced not only by main effects but also by combined demographic and geographic modifiers. Wald tests further indicated that age (χ^2^ = 4.93, *p* = 0.0264) and region (χ^2^ = 476.81, *p* < 0.001) were significant determinants of specificity. Residual heterogeneity was minimized in this full interaction model (τ^2^ = 0.00 for sensitivity; τ^2^ = 0.01 for specificity), with the LR test for heterogeneity (χ^2^ = 13.27, *p* = 0.0041) confirming improved fit. These findings indicate that variation in specificity is partly explained by higher order interactions, whereas sensitivity remains largely unaffected.

#### Post hoc analysis of cut-off and ELISA method

Post hoc analyses were conducted to assess whether threshold definitions or ELISA methodology accounted for variation in diagnostic performance (Table 7). When cut-off parameters were examined independently, sensitivity differed significantly across thresholds (χ^2^ = 4.31, *p* = 0.04), while specificity remained stable (χ^2^ = 0.25, *p* = 0.62). Pooled estimates showed that the 2SD threshold yielded a sensitivity of 0.65 (95% CI: 0.43–0.82) and specificity of 0.89 (95% CI: 0.74–0.96), whereas the 3SD threshold demonstrated higher sensitivity 0.87 (95% CI: 0.79–0.92) and comparable specificity 0.92 (95% CI: 0.85–0.95).

**Table 7:** Multivariate independent meta-regression of αvβ6 diagnostic accuracy by type of control, cut-off parameters, Elisa method and region

Variable	Sensitivity			Specificity		
	Chi^2^	df	*p* value	Chi^2^	df	*p* value
*Meta-regression by cut-off parameters*						
Cut-off parameter (+2SD from mean as standard)	4.31	1	0.04	0.25	1	0.62
*Meta-regression by ELISA method*						
ELISA (Kuwada method as standard)	0.21	1	0.65	1.30	1	0.25
*Meta-regression by type of control and cut-off parameters*						
Control (healthy control as standard)	4.17	1	0.04	0.24	1	0.63
Cut-off parameter (+2SD from mean as standard)	0.18	3	0.98	25.26	3	< 0.001
Full model	4.60	4	0.33	25.51	4	< 0.001
*Meta-regression by type of control and ELISA method*						
Control (healthy control as standard)	0.22	1	0.64	0.26	1	0.61
ELISA method (method used and referenced by Kuwada as standard)	0.19	3	0.98	22.35	3	< 0.001
Full model	0.40	4	0.98	23.65	4	< 0.001
Meta-regression by region and cut-off parameters						
Not estimable (perfect collinearity between region and cut-off; structural empty cells)^1^						
Meta-regression by region and ELISA method						
Region (Europe as standard)	0.10	1	0.75	0.83	1	0.36
ELISA method (method used and referenced by Kuwada as standard)	6.48	2	0.04	7.22	2	0.03
Full model	6.70	3	0.08	8.53	3	0.04

*p*-values <0.05 were considered statistically significant

^1^Not estimable**:** model could not be fitted due to perfect/near-perfect collinearity between *region* and *cut-off rule* (all European studies used +2SD/95th-percentile thresholds; no within-region variability). This produced structural empty cells, so convergence was not achievable. We, therefore, report region and cut-off effects in separate models and via restriction analyses

In contrast, ELISA platform alone did not show significant associations with either sensitivity (χ^2^ = 0.21, *p* = 0.65) or specificity (χ^2^ = 1.30, *p* = 0.25). Descriptively, Kuwada-based assays had pooled sensitivity of 0.82 (95% CI: 0.72–0.90) and specificity of 0.92 (95% CI: 0.86–0.95), while non-Kuwada platforms (Okabe 2024) demonstrated slightly higher sensitivity 0.88 (95% CI: 0.58–0.97) but lower specificity 0.83 (95% CI: 0.59–0.94).

In multivariable models combining control type with cut-off parameters, sensitivity was no longer influenced by cut-off parameters (χ^2^ = 0.18, *p* = 0.98), whereas specificity varied significantly (χ^2^ = 25.26, *p* < 0.001). The full model confirmed this effect (χ^2^ = 25.51, *p* < 0.001). Similarly, when control type was modeled with ELISA method, specificity again differed significantly by platform (χ^2^ = 22.35, *p* < 0.001), while sensitivity remained unaffected (*p* = 0.98). Attempts to jointly model region and cut-off parameters failed to converge due to perfect collinearity, as all European studies exclusively applied +2SD or 95th percentile thresholds. However, when region and ELISA method were modeled together, region showed no significant effect on sensitivity (χ^2^ = 0.10, *p* = 0.75) or specificity (χ^2^ = 0.83, *p* = 0.36), whereas ELISA method remained significant for both sensitivity (χ^2^ = 6.48, *p* = 0.04) and specificity (χ^2^ = 7.22, *p* = 0.03). Importantly, residual heterogeneity decreased markedly in this model (generalized τ^2^ = 0.05 compared with τ^2^ = 0.27 in unadjusted ELISA models).

Taken together, these results indicate that apparent regional differences in specificity are largely explained by methodological factors, particularly the choice of assay platform and cut-off definition, while sensitivity remains robust across analytic specifications.

## Discussion

Our meta-analysis demonstrated that anti-integrin αvβ6 antibodies possess high pooled sensitivity (83%) and specificity (93%) for diagnosing ulcerative colitis, indicating strong overall diagnostic performance across diverse patient cohorts. Sensitivity remained consistent across control subgroups and was unaffected by age group or geographic region in meta-regression models, suggesting potential generalizability in identifying true UC cases. Specificity, by contrast, varied significantly by control group type, patient age, and geographic region, as demonstrated by both univariate and multivariable meta-regression. Specificity was lowest when Crohn’s disease served as the comparator group, consistent with the immunological overlap between UC and CD. Similarly, lower specificity in pediatric and U.S. cohorts may reflect age-related immunologic variability or regional assay performance differences, though the exact mechanisms remain unclear. While our study did not assess disease epidemiology directly, the observed regional and age-related variability in diagnostic performance underscores the need to consider demographic factors when interpreting serologic results [19]. These differences in specificity suggest that diagnostic accuracy may be context-dependent, potentially influenced by population-level factors such as baseline disease prevalence or assay calibration. The high heterogeneity observed initially in the present study may also be accounted for by variations in age groups and geographical regions. The diagnosis of IBD involves the use of invasive and non-invasive tests, markers and interventions, with variable diagnostic accuracies [20]. Endoscopy remains the primary diagnostic modality in IBD, with guidelines recommending 2 biopsy segments from at least 5 sites for diagnostic purposes [21]. It allows visualization of gastrointestinal mucosa and involvement of various regions such as terminal ileum and rectum, with a pivotal role not only in diagnosis but also in the evaluation of disease activity and progression [22]. However, it is an invasive test which can be uncomfortable and painful for the patient [20], thus it is recommended only in the presence of a high deal of suspicion, leading to a delay in diagnosis of an average 12 months in UC [20]. In addition, associated complications include endoscope-associated infections (EAI), bleeding, perforation, cholangitis and pancreatitis [23], further creating reluctance on the part of the patient.

Inflammatory markers that have been reported to aid in diagnosis of IBD include calprotectin, cytokines such as IL-6, IFN-γ, and MIP-1β, salivary amylase, mucin 5B, salivary IgA, cortisol, C reactive protein (CRP), and stool lactoferrin [24, 25]. However, these markers possess limited diagnostic accuracy. A meta-analysis conducted by Mosli et al. reveals a pooled sensitivity and specificity of 49% and 92% for CRP, 88% and 73% for fecal calprotectin, and 82% and 79% for lactoferrin, respectively [24]. In a study by Duvoisin et al., MPO showed high sensitivity (89%) but low specificity (51.4%) for UC patients compared to no inflammation [26]. Fecal MPO levels were significantly higher in UC patients than in healthy controls (0.42 units vs 0.06 units; *p* < 0.001) [26]. p-ANCA and ASCA have been identified as useful biomarkers for distinguishing CD from UC [27]. p-ANCA was more commonly seen in UC and colonic CD, while ASCA was more associated with CD (sensitivity 100%, specificity 50%) [28]. A meta-analysis showed that the ASCA+/p-ANCA− pattern had high specificity (0.93) but moderate sensitivity (0.55 for CD, 0.63 for IBD) [27], making it more useful for identification of CD [29]. This indicates that anti-αvβ6 may represent a reliable diagnostic biomarker for UC compared with existing serologic markers. We recommend that future studies should utilize the combination of avB6 with established serological markers such as p-ANCA to further investigate its effect on diagnostic yield.

In addition, studies are present outlining raised interleukin levels in UC and their role in monitoring disease progression; however, their role in primary diagnostics remains relatively unexplored [30]. Similarly, data on salivary amylase for UC are limited [31].

Our findings indicate that αvβ6 antibodies may offer a diagnostic performance that is at least comparable and in some subgroups, superior to currently available non-invasive markers. The biological relevance of αvβ6 in epithelial barrier integrity and immune modulation further supports its utility as a disease-specific biomarker [7].

Our post hoc analyses showed that the regional differences observed in specificity were caused by methodological factors, especially the ELISA kit used and cut-off definition/value. While sensitivity was generally stable across the groups, specificity varied depending upon the comparative group, i.e., studies using Crohn’s disease as the comparator showed the greatest decrease. Importantly, once assay method and cut-off definition were analyzed in multivariable models, most of the regional variation disappeared and overall heterogeneity declined. This indicated that these variables caused much of the apparent regional effect rather than true biological variation. These findings suggest that αvβ6 sensitivity is consistent across cohorts, while specificity is context-dependent and should be interpreted considering study methodology.

To our knowledge, this is the first meta-analysis to systematically evaluate the diagnostic accuracy of anti-integrin αvβ6 antibodies in ulcerative colitis, incorporating meta-regression to assess the influence of demographic and regional factors on test performance. The use of bivariate and multivariable models allowed for structured examination of heterogeneity and identified age and geographic region as significant modifiers of diagnostic specificity. These findings underscore the importance of contextual variables when evaluating novel serologic biomarkers for inflammatory and autoimmune diseases. Subgroup analyses further reinforced the robustness of the primary results across relevant clinical comparators. Advanced meta-regression analyses were performed to evaluate the impact of key covariates such as control group type, age, and geographic region on diagnostic performance. These analyses enhanced the robustness of the findings by identifying important factors influencing specificity and providing a deeper understanding of the variability in αvβ6 antibody accuracy across diverse populations. Taken together, our findings support the diagnostic potential of anti-integrin αvβ6 antibodies in ulcerative colitis, particularly as a non-invasive serologic tool with high sensitivity and specificity. The stability of sensitivity across diverse populations and the identification of key moderators of specificity provide important context for clinical application and future assay development. A key strength of this study is the post hoc analysis, which identified the impact of different cut-off thresholds and ELISA platforms on specificity, further refining the understanding of αvβ6 antibody performance in UC diagnosis. Further prospective studies are warranted to validate these findings in broader and more diverse cohorts, standardize assay protocols, and explore the utility of αvβ6 in preclinical detection and longitudinal disease monitoring.

This meta-analysis is limited by the small number of eligible studies evaluating anti-integrin αvβ6 for the diagnosis of ulcerative colitis, which constrains the statistical power of subgroup and meta-regression analyses and limits the ability to detect subtle effect modifiers. Although study quality was generally high, incomplete reporting of key covariates such as smoking status, medication use, and disease severity restricted adjustment for potential confounding factors and limited insight into biomarker performance across clinically relevant subgroups. Initiation or tapering of immunosuppressive therapy could alter antibody production and circulating titers, which can influence diagnostic accuracy [32]. Most included studies did not provide information regarding prior drug history of patients such as immunosuppressants, corticosteroids, or biologic therapies, potentially confounding the diagnostic accuracy estimates. Marked heterogeneity in specificity estimates was observed across studies. While control group type, geographic region, and patient age explained some of this variability, additional sources of heterogeneity likely include inter-study differences in ELISA platforms, cut-off thresholds, and sample processing protocols. While our use of a bivariate random-effects model approximately accounts for such threshold heterogeneity, this methodological variability may affect reproducibility and limit direct comparison across cohorts. For the region and cut-off value meta-regression, model could not be fitted due to near-perfect collinearity between *region* and *cut-off rule*: all European studies used +2SD/95th-percentile thresholds, leaving no within-region variability. This resulted in structural empty cells, so convergence was not achievable. We, therefore, analyzed region and cut-off effects separately. To establish a standardized cut-off, more studies with consistent reporting are required. The geographic scope of included studies was confined to Europe, Asia, and North America, with minimal data available from underrepresented regions such as Africa, South America, or the Middle East. Furthermore, race and ethnicity data were not consistently reported, precluding assessment of biomarker performance across diverse populations. Finally, due to the small number of included studies, formal testing for publication bias could not be performed. As a result, selective reporting of positive findings cannot be excluded.

### Conclusion

Anti-integrin αvβ6 antibodies demonstrate high diagnostic accuracy for ulcerative colitis, with robust sensitivity and specificity across diverse comparator groups. Sensitivity remained stable across age groups, geographic regions, and control types, while specificity was influenced by population demographics and clinical context. These findings position αvβ6 as a promising non-invasive serologic biomarker for UC, with potential utility in early diagnosis, risk stratification, or as an adjunct to endoscopic evaluation. However, assay heterogeneity and limited population diversity across studies warrant further prospective validation in standardized platforms and globally representative cohorts before routine clinical adoption.

## Supplementary Information

Below is the link to the electronic supplementary material.

Supplementary file1 (DOCX 19 kb)

## Abbreviations

UC: Ulcerative colitis
IBD: Inflammatory bowel disease
CD: Crohn’s disease
GI: Gastrointestinal
AUC: Area under curve
CI: Confidence interval
TP: True positive
FP: False positive
TN: True negative
FN: False negative
