# Supplementary material for: Diagnostic accuracy of anti-integrin αvβ6 in ulcerative colitis: a diagnostic meta-analysis
Source: J Gastroenterol. 2025 Nov 13;61(1):1–15. doi: 10.1007/s00535-025-02319-8 (PMC12791060; doi:10.1007/s00535-025-02319-8)
Supplement: Supplementary file 1 — Supplementary file1 (DOCX 19 kb) [file 535_2025_2319_MOESM1_ESM.docx]

**Supplementary Table 1:** **Heterogeneity from Independent Meta-regression Models**
Summarizes between-study heterogeneity and model fit for control type, age, region, cut off parameters and ELISA method analyzed independently and in multivariable models. Results show each covariate's contribution to heterogeneity in sensitivity and specificity.

| **Source of Heterogeneity** | **Between study heterogeneity** | | | **LR test: RE vs. FE model** | |
| --- | --- | --- | --- | --- | --- |
|  | **Tau square** | | |  |  |
|  | **Generalized** | **Sensitivity** | **Specificity** | **Chi^2^** | **p value** |
| ***Univariate analysis by type of control (Table 4)*** | 0.15 | 0.69 | 0.23 | 121.98 | **<0.001** |
| ***Multivariate model: Control + Age (Table 5)*** | 0.00 | 0.45 | 0.00 | 88.18 | **<0.001** |
| ***Multivariate model: Control + Region (Table 5)*** | 0.00 | 0.27 | 0.07 | 32.97 | **<0.001** |
| ***Univariate analysis by cut off parameter (Table 6)*** | 0.15 | 0.39 | 0.49 | 70.94 | **<0.001** |
| ***Univariate analysis by ELISA method (Table 6)*** | 0.27 | 0.70 | 0.39 | 148.86 | **<0.001** |
| ***Multivariate model: Control + Cut off parameter (Table 6)*** | 0.00 | 0.36 | 0.16 | 57.16 | **<0.001** |
| ***Multivariate model:***  ***Control + ELISA method (Table 6)*** | 0.15 | 0.67 | 0.24 | 100.45 | **<0.001** |
| ***Multivariate Model:  Region + ELISA method (Table 6)*** | 0.05 | 0.28 | 0.17 | 34.15 | **<0.001** |

**Supplementary Table 2:** **Heterogeneity from Interaction Meta-regression Models**
Reports heterogeneity estimates from models including interaction terms (e.g., control × age × region). The multivariable interaction model notably reduced residual heterogeneity, highlighting significant effect modification.

| **Source of Heterogeneity** | **Between study heterogeneity** | | | **LR test: RE vs. FE model** | |
| --- | --- | --- | --- | --- | --- |
|  | **Tau square** | | |  |  |
|  | **Generalized** | **Sensitivity** | **Specificity** | **Chi^2^** | **p value** |
| ***Control vs. Crohns (Table 7)*** | 0.16 | 0.72 | 0.26 | 193.07 | **<0.001** |
| ***Age (Table 7)*** | 0.00 | 0.48 | 0.00 | 146.95 | **<0.001** |
| ***Region (Table 7)*** | 0.02 | 0.28 | 0.12 | 36.61 | **<0.001** |
| ***Multivariable Metaregression (Table 7)*** | 0.00 | 0.20 | 0.01 | 13.27 | **0.0041** |
